# The Novel Disease *Vicia unijuga* Caused by *Colletotrichum tofieldiae* in China: Implications for Host Growth, Photosynthesis, and Nutritional Quality

**DOI:** 10.3390/jof11080567

**Published:** 2025-07-29

**Authors:** Tong-Tong Wang, Hang Li, Yan-Zhong Li

**Affiliations:** State Key Laboratory of Herbage Improvement and Grassland Agro-Ecosystems, Engineering Research Center of Grassland Industry, College of Pastoral Agriculture Science and Technology, Lanzhou University, Lanzhou 730020, China; wangtt2023@lzu.edu.cn (T.-T.W.); lzucookie@163.com (H.L.)

**Keywords:** perennial vetch, anthracnose, host range, growth indicators, photosynthesis

## Abstract

*Vicia unijuga*, an important forage legume on China’s Qinghai–Tibetan Plateau, exhibited dark-brown sunken lesions on their stems at the Qingyang Experimental Station of Lanzhou University. The fungus isolated from the diseased tissues was identified as *Colletotrichum tofieldiae* via a multi-locus phylogeny (ITS-*ACT*-*Tub2*-*CHS-1*-*GADPH*-*HIS3*). The pathogenicity was confirmed by Koch’s postulates. The inoculated plants showed significantly reduced (*p* < 0.05) growth parameters (height, root length, and biomass), photosynthetic indices (net rate, transpiration, and stomatal conductance), and nutritional quality (crude protein, crude fat, crude ash, and crude fiber) compared to the controls. *C. tofieldiae* additionally infected six legume species (*V. sativa*, *Medicago sativa*, *Onobrychis viciifolia*, *Astragalus adsurgens*, *Trifolium pratense*, and *T. repens*). Optimal in vitro growth occurred on oatmeal agar (mycelium) and cornmeal agar (spores), with D-sucrose and D-peptone as the best carbon and nitrogen sources. This first report of *C. tofieldiae* causing *V. unijuga* anthracnose advances the understanding of legume anthracnose pathogens.

## 1. Introduction

*Vicia unijuga* (commonly known as perennial vetch), an important forage legume in the Leguminosae family, is broadly distributed in China, North Korea, and Russia [1]. *V. unijuga* has an extremely extensive utilization value as a medicinal plant, edible wild vegetable, and landscaping plant. Nonetheless, its feed value is most important because of its high protein and soluble sugar content [2]. However, the occurrence of diseases adversely affects the plant growth, yield, and nutritional quality of *V. unijuga*. Six genera, including *Cylindrosporium* [3], *Erysiphe* [4,5,6], *Microsphaera* [4], *Oidium* [4,6], *Septoriella* [7], and *Uromyces* [4,5,8,9,10], cause fungal diseases in *V. unijuga* [11]. Of note, five of these fungal pathogens have been reported in China.

*Colletotrichum* (*Glomerellaceae*, *Glomerellales, Sordariomycetes,* and *Ascomycota*) is a large genus of ascomycete fungi that comprises 280 species, whose molecular data is available and accepted in the genus [12]. The *Colletotrichum* species include pathogens, endophytes, and saprobes. Most of the *Colletotrichum* pathogens are the principal causes of plant anthracnose in almost every crop grown globally [13]. More than 97% of the documented *Colletotrichum*–host associations occur on dicotyledonous and monocotyledonous plants [14], whereas 33 species are endophytes that have mutualistic relationships with their hosts [12,15]. Notably, the *Colletotrichum* pathogens are the eighth most important group of plant pathogenic fungi globally, based on their scientific and economic importance [13]. These pathogens can infect the stems, leaves, flowers, and fruits, which hinders plant growth and reduces yield [16]. The *Colletotrichum* species can infect multiple *Vicia* species. For instance, *V. faba* has been infected by *C. acutatum* in Australia [17], while *C. lentices* and *C. spinaciae* have caused anthracnose in *V. sativa*, which has led to serious losses in the Gansu province of China [18,19,20].

Previously, *Colletotrichum* was classified based on its morphology and host range. However, there are only a few unique morphological characteristics that can be used for identification, which has led to the misidentification of species because of the lack of a clear understanding of host specificity [21]. In recent years, a multi-locus sequence analysis has become the primary method for accurate species identification [22]. Many researchers have provided a comprehensive set of methods to guide the delimitation and classification of *Colletotrichum* species [21,23]. Currently, the internal transcribed spacers (ITS), glyceraldehyde-3-phosphate dehydrogenase (*GAPDH*) gene, and actin (*ACT*) gene are the commonly used genes for the molecular identification of *Colletotrichum*. Both existing and newly discovered *Colletotrichum* species can be classified and identified based on a multi-locus sequence analysis of these genes [24,25].

*V. unijuga* is a wild forage legume widely distributed in the alpine steppe of the Qinghai–Tibetan Plateau of China. It has been selected as a cultivated forage legume for breeding because of its strong adaptability [26,27,28], good palatability, and high feeding value [2]. Noteworthy, the research team from the College of Pastoral Agriculture Science and Technology of Lanzhou University successfully domesticated and selected cultivated *V. unijuga* varieties after years of experimentation, which have since been approved. Comprehensively studying plant diseases is foundational for large-scale cultivation and is critical for developing effective disease control strategies.

Currently, the pathogen responsible for anthracnose in *V. unijuga* remains poorly understood. This study thus aimed to (i) identify the causal pathogen through tissue isolation, morphological observation, pathogenicity, and a multi-locus sequence analysis; (ii) explore the host range and biological characteristics of the causal pathogen; and (iii) study its effects on the growth and photosynthesis indicators and nutritional quality of *V. unijuga*.

## 2. Materials and Methods

### 2.1. Sampling and Isolation

*V. unijuga* plants exhibiting disease symptoms on their stems were identified and collected from the Qingyang Experimental Station of Lanzhou University (35.67° N, 107.85° E) in Gansu Province in September 2022. The stem tissues of the *V. unijuga* plants at the border between the symptomatic and healthy areas were selected for pathogen isolation. The stem surfaces were first rinsed with running water, disinfected with 75% alcohol for 15 s and 1% NaClO for 3 min, washed thrice with sterile water, and then dried using sterile filter paper. The stem tissues were subsequently cut into appropriate sizes, followed by evenly plating five pieces onto potato dextrose agar (PDA, Beijing Land Bridge Technology Co., Ltd., Beijing, China). Stem sampling was conducted using ten plants. The tissues from each plant were plated onto 3 PDA plates. Fresh mycelium was picked and purified on PDA after the initial colony formation. The purified isolates were stored at 4 °C and −80 °C in the Strain Preservation Library of the College of Pastoral Agriculture Science and Technology of Lanzhou University.

### 2.2. Morphological Characterization

Fungal plugs were grown on PDA medium at 25 °C to observe colony morphology and measure their growth rates. Micrographs of conidia and sporodochia, 100 and 50, respectively, were randomly taken. Moreover, 50 conidial appressoria [29] were randomly measured. Scanning electron microscope (Apreo S, Thermo Fisher Scientific, Waltham, MA, USA) was used for observations and measurements.

### 2.3. DNA Extraction, PCR Amplification, Sequencing, and Phylogenetic Analysis

Fungal DNA was extracted from fresh mycelium using an Omega Biotek E.Z.N.A^®^ High Performance (HP) Fungal DNA Kit (Norcross, GA, USA). The fungal isolates were further characterized by amplifying six gene loci: ITS using ITS1/ITS4 primers [30], *ACT* using ACT-512F/ACT-783R primers [31], β-tubulin (*Tub2*) using T1/Bt-2b primers [32,33], chitin synthase 1 (*CHS-1*) using CHS-354R/CHS-79F primers [31], *GAPDH* using GDF1/GDR1 primers [34], and histone H3 (*HIS3*) using CYLH3F/CYLH3 primers [35]. The primers used were synthesized by Sangon Biotech Co., Ltd., Shanghai, China.

A polymerase chain reaction (PCR) was performed using a BIO-RAD T100 thermal cycler. The reaction mix comprised 12.5 μL of 2 × Taq PCR Master Mix (Tiangen Biotech Co., Ltd., Beijing, China), 9.5 μL of double-distilled H_2_O, 1 μL of each primer, and 1 μL of template DNA, totaling 25 μL. The PCR amplification program was as follows: an initial denaturation at 95 °C for 30 s; primer annealing at 56 °C (ITS), 59 °C (*GADPH*), 57 °C (*ACT*), 56 °C (*Tub2*), 59 °C (*CHS-1*), and 57 °C (*HIS3*) for 30 s, and extension at 72 °C for 1 min; and a final extension at 72 °C for 10 min. The PCR products were sent to Sangon Biotech (Shanghai) Co., Ltd. for bidirectional sequencing.

The sequence reads were subsequently spliced using SeqMan 5.0 (DNASTAR, Inc^®^. Madison, WI, USA) and blasted in the National Center for Biotechnology Information (NCBI) database to find regions of local similarity between the sequences. A phylogenetic tree construction was performed using carefully selected type strain sequences (ex-type/ex-epitype) of the target species, validated by Damm et al. [36] and retrieved from GenBank (Table S1). Both the individual and multi-locus genes were aligned and trimmed using MEGA 6.0 software [37]. A multi-gene phylogenetic tree was constructed using the maximum likelihood (ML) method [38] using MEGA 6.0. The tree was based on the concatenated sequences from six genomic regions (ITS-*ACT*-*Tub2*-*CHS-1*-*GAPDH*-*HIS3*), with *C. lindemuthianum* used as the outgroup.

### 2.4. Koch’s Postulates and Host Range

Seven common leguminous forages, *V. unijuga*, *V. sativa*, *Medicago sativa*, *Onobrychis viciifoila*, *Astragalus adsurgens*, *Trifolium pratense*, and *T. repens*, were selected to assess the pathogenicity of the fungus in the greenhouse. The forage species were grown for two weeks, and their stems were subsequently sprayed with a conidial suspension of the isolate LYZ0664 (approximately 10^6^ CFU mL^−1^) [39] and kept in a moist environment at 25 °C. The control plants were sprayed with sterile water. Each forage species had 45 treated and 45 control plants. The plants were covered for 48 h with black plastic bags sprayed with sterile water to create a moist environment for infection to take place.

The disease progression was monitored and recorded, and subsequent statistical calculations of the incubation period, incidence rate (1), disease index (2), and reisolated rate (3) for each plant species were performed. The incubation period means the time elapsed from pathogen inoculation to the first observation of typical disease symptoms (measured in days). The disease severity was classified based on a slightly modified Li and Nan’s [40] disease severity standard (Table S2).

(1) $$\mathrm{incidence}\;\mathrm{rate}\;(\%)=\frac{\mathrm{diseased}\;\mathrm{palnts}}{\mathrm{total}\;\mathrm{number}\;\mathrm{of}\;\mathrm{investigated}\;\mathrm{plants}}\times 100,$$

(2) $$\mathrm{disease}\;\mathrm{index}=\frac{\sum \left(\mathrm{number}\;\mathrm{of}\;\mathrm{plants}/\mathrm{scale}\;\times \;\mathrm{scale}\;\mathrm{value}\right)}{\left(\mathrm{highest}\;\mathrm{scale}\;\mathrm{vale}\;\times \;\mathrm{total}\;\mathrm{number}\;\mathrm{of}\;\mathrm{plants}\right)}\times 100,$$

(3) $$\mathrm{reisolated}\;\mathrm{rate}\;(\%)=\frac{\mathrm{number}\;\mathrm{of}\;\mathrm{diseased}\;\mathrm{plants}\;\mathrm{reisolated}\;\mathrm{to}\;\mathrm{the}\;\mathrm{target}\;\mathrm{pathogen}}{\mathrm{total}\;\mathrm{number}\;\mathrm{of}\;\mathrm{diseased}\;\mathrm{plants}}\times 100,$$

### 2.5. Biological Characteristics of Isolated Fungi

The isolated fungi were grown on various media, carbon and nitrogen sources, and at different temperatures to determine how their growth rates and sporulation were affected by these changes. Ten types of media were tested: PDA, corn meal agar (CMA), potato carrot agar (PCA), oatmeal agar (OMA), potato sucrose agar (PSA), carnation leaf agar (CLA), water agar (WA), wheat straw decoction agar (WHDA), beef extract peptone agar (NA), and tomato agar (V8). Eight types of carbon sources were tested in 5 g KNO_3_, 2 g Na_3_PO_4_, 1 g Mg_2_SO_4_, 17 g agar, and 1000 mL distilled water as the basic medium. The carbon sources were 20 g of glucose, fructose, sucrose, lactose, starch-soluble, maltose, xylose, and mannitol. Seven types of nitrogen sources were tested in 5 g glucose, 2 g Na_3_PO_4_, 1 g Mg_2_SO_4_, 17 g agar, and 1000 mL distilled water as the basic medium. The nitrogen sources were 5 g of peptone, urea, NaNO_3_, (NH_4_)_2_SO_4_, aspartate, tryptophan, and glycine. Seven temperatures were tested: 5 °C, 10 °C, 15 °C, 20 °C, 25 °C, 30 °C, and 35 °C. The tests on the different culture media, carbon, and nitrogen sources were performed at 25 °C in the dark. The different temperatures were tested using PDA medium.

Hyphal plugs of the isolate (diameter = 0.5 cm) were inoculated at the center of the culture dishes, with 5 replicates per treatment. The colony diameter was measured using a vernier caliper after 7 days of culture.

The sporulation was assessed using a hemocytometer (25 middle squares × 16 small squares per counting chamber, chamber volume = 0.1 mm^3^; Anhui Ranjeck Technology Co., Ltd., Hefei, China) after 21 days of fungal growth. To each fungal culture plate, 5 mL of sterile distilled water was added, and the mycelial surface was gently scraped with a sterile spreader to obtain spore suspension. The resulting suspension was filtered through four layers of sterile gauze to remove mycelial debris, and the filtrate was collected in a sterile centrifuge tube. The spores were pelleted by centrifugation and resuspended in sterile water to an appropriate dilution. A calibrated volume of the diluted spore suspension was pipetted onto the hemocytometer, and the spores within the five middle squares (selected via the five-point sampling method) were counted under a light microscope using the “count top and left, exclude bottom and right” rule. This counting process was repeated 5 times per sample. The spore concentration was calculated using Formula (4).

(4) $$\mathrm{Sporulation}\;\mathrm{concentration}\;(\mathrm{CFU}/\mathrm{mL})=\frac{\mathrm{total}\;\mathrm{spores}\;\mathrm{in}\;5\;\mathrm{middle}\;\mathrm{squares}}{5}\times 25\times 10,000\times \mathrm{dilution}\;\mathrm{multiple}$$

### 2.6. Influence of the Isolate LYZ0664 on the Growth Indicators of V. unijuga

The growth indicators of *V. unijuga* were measured on the 21st day post inoculation with the isolate LYZ0664. The plant height was measured from the soil surface to the plant’s highest point. The dry weight of this section was recorded as the aboveground dry weight. The root length extended from the base of the plant to the root tip. The dry weight of this section was recorded as the underground dry weight.

### 2.7. Influence of the Isolate LYZ0664 on the Photosynthesis Indicators of V. unijuga

The photosynthetic parameters of *V. unijuga*, such as the photosynthetic rate (Pn), stomatal conductance (Gs), intercellular carbon dioxide concentration (Ci), and transpiration rate (Tr), were measured on the 7th, 14th, and 21st days post inoculation with the isolate LYZ0664. These measurements were performed using a GFS-3000 gas exchange system (WALZ). The measurements were taken for the same leaf position with consistent growth and illumination across 5 plants. The measurements were performed for 3 leaves per plant and each leaf was measured thrice.

### 2.8. Influence of the Isolate LYZ0664 on the Nutritional Quality of V. unijuga

Thirty-five diseased and control plants of *V. unijuga* were sampled from the greenhouse and sent to the Forage Quality Testing Center of the Feeding Laboratory of Lanzhou Polison Ecological Science and Technology Co., Ltd., Lanzhou, China in Gansu Province for a conventional nutrient analysis. The nutritional components analyzed included the crude protein, crude ash, crude fat, neutral detergent fiber, acid detergent fiber, crude fiber, and water content [41].

### 2.9. Data Analysis

A statistical analysis of the various parameters was performed using SPSS 22.0, while Duncan’s method was used for significance testing. The results are expressed as means ± standard error (SE). Photo editing was performed using GraphPad Prism 8 and Photoshop CS6.

## 3. Results

### 3.1. Disease Survey and Strain Isolation

**Figure 1:**
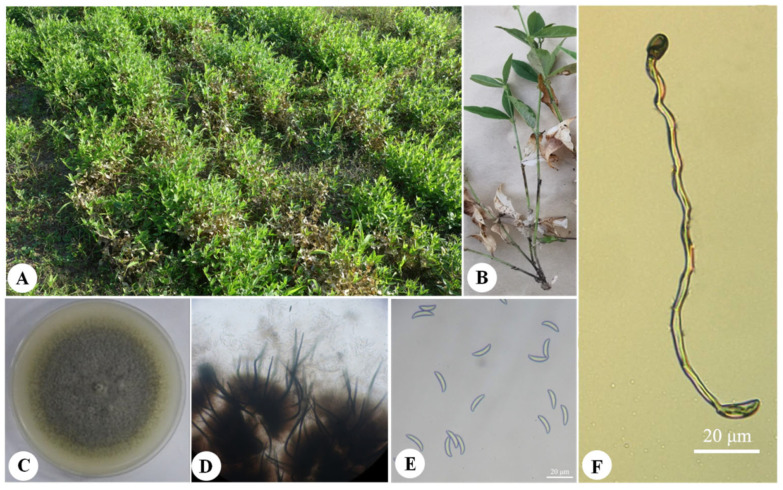
Field symptoms and morphological characteristics of *Colletotrichum tofieldiae* (LYZ0664). (**A**,**B**) Field symptoms. (**C**) Colony on PDA after 7 days at 25 °C. (**D**) Acervuli and setae. (**E**) Conidia on PDA. (**F**) Appressorium.

A disease survey in the field revealed a disease incidence rate of 78.93% and a disease index of 57.77. The susceptible plants exhibited complete desiccation and death (Figure 1A). The lesions on the stems were linear and dark brown, expanding to a diameter of 1 to 4 cm (Figure 1B). The potential pathogenic fungus was isolated, with a plant carrying rate of 100% and an isolation rate of 46.67%. Three strains with the same cultural characteristics of the fungus were selected for purification and were numbered as LYZ0664, LYZ0665, and LYZ0666.

### 3.2. Morphological Characterization of LYZ0664

The colony color on the PDA medium was pink-white initially, with black filamentous hyphae appearing at the center after 3 days. The colony color then turned gray-green and eventually became black with gray-white protrusion particles on the surface. The short, dense hyphae grew in a wheel-like pattern (Figure 1C). Conidiomata brown, with setae, directly formed on them. Setae brown, septate, smooth, 97.2 to 151.2 μm long, and were conically enlarged at the base and acutus at the top (Figure 1D). Conidia hyaline, crescent shaped, aseptate, smooth walled, and 15.27 to 19.73 × 3.66 to 5.72 μm in size (Figure 1E). Conidial appressoria, solitary, ellipsoidal to clavate, smooth, aseptate, brown or dark brown to almost black, and 8.5 to 21.5 × 4.5 to 11 μm in size (Figure 1F).

### 3.3. Phylogenetic Analysis

**Figure 2:**
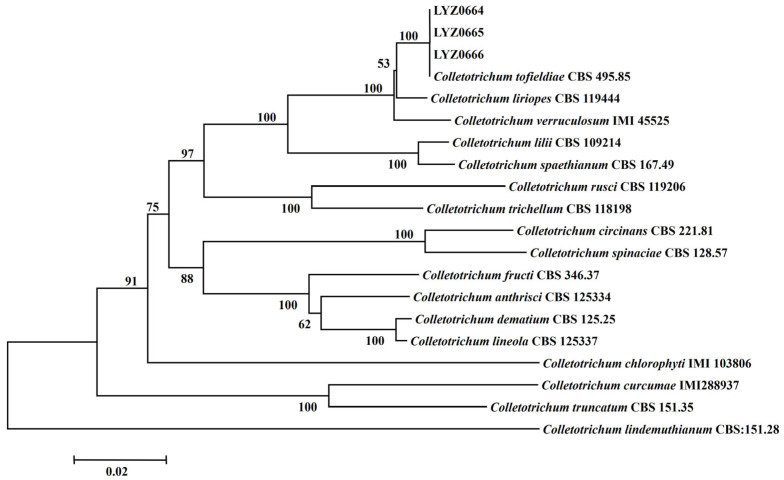
Maximum likelihood (ML) phylogenetic analysis of concatenated ITS, *ACT*, *Tub2*, *CHS-1*, *GAPDH*, and *HIS3* sequences reveals the clustering patterns of *Colletotrichum tofieldiae* strains obtained from *Vicia unijuga*. GenBank accession numbers are shown in Table S1.

The BLAST analysis of the ITS, *ACT*, *Tub2*, *CHS-1*, *GAPDH*, and *HIS3* gene sequences of LYZ0664, LYZ0665, and LYZ0666 using the NCBI database revealed the high similarity (>99.8%) of the six DNA sequences to *C. tofieldiae*. LYZ0664, LYZ0665, LYZ0666, and *C. tofieldiae* (CBS 495.85) clustered on the same branch, demonstrating a 100% genetic relationship (Figure 2). The strains were identified as *C. tofieldiae* based on a multi-locus phylogenetic analysis.

### 3.4. Koch’s Postulates and Host Range of LYZ0664

**Figure 3:**
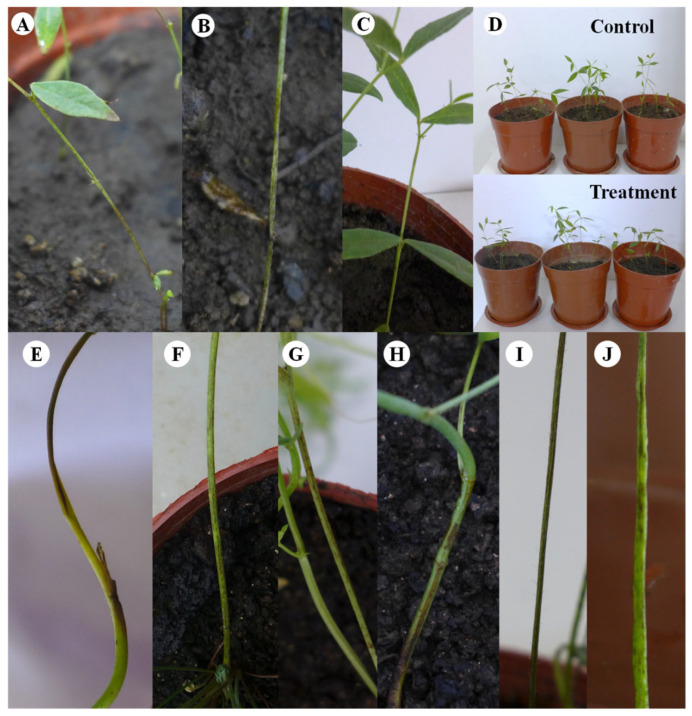
Inoculation symptoms. (**A**–**D**) Koch’s postulates of *Colletotrichum tofieldiae* on *Vicia unijuga*. (**A**,**B**) Symptoms of inoculation. (**C**) Control. (**D**) Control and infected potted plants. (**E**–**J**) Inoculation symptom of other six legumes: (**E**) *Medicago sativa*; (**F**) *Onobrychis viciifoila*; (**G**) *V. sativa*; (**H**) *Astragalus adsurgens*; (**I**) *Trifolium pretense*; (**J**) *T. repens*.

Small, round, yellowish-brown, and sunken lesions manifested on the stems of *V. unijuga* 4 days post inoculation (Figure 3A). The lesions spread into a single mass, with the stems progressively turning black on the 7th day (Figure 3B). In contrast, the control remained unaffected (Figure 3C). Noteworthy, the inoculated plants exhibited reduced vigor (Figure 3D).

The inoculation of *V. sativa*, *M. sativa*, *O. viciifolia*, *A. adsurgens*, *T. pratense*, and *T. repens* with the pathogenic fungus under greenhouse conditions led to all six forages displaying similar symptoms. Small black-brown lesions appeared during the early stage, which subsequently expanded into larger patches (Figure 3E–J).

*C. tofieldiae* infected all seven tested legume forages, with the infection severity varying among the hosts. Notably, the highest incidence rate (73.33%) and disease index (57.22) (Table 1) were observed on *V. sativa*, which belongs to the same genus as the pathogen’s original host.

### 3.5. Biological Characteristics of LYZ0664

**Figure 4:**
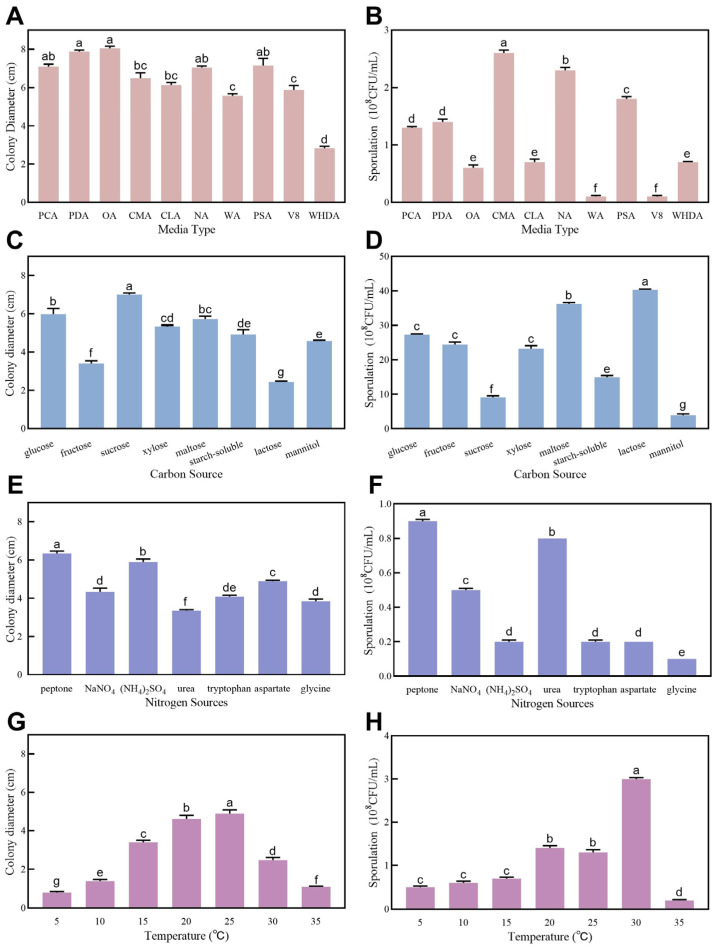
Colony growth and sporulation of *Colletotrichum tofieldiae* under different media types, carbon sources, nitrogen sources, and temperatures. (**A**) Effects of media types on colony growth; (**B**) Effects of media types on sporulation; (**C**) Effects of carbon sources on colony growth; (**D**) Effects of carbon sources on sporulation; (**E**) Effects of nitrogen sources on colony growth; (**F**) Effects of nitrogen sources on sporulation; (**G**) Effects of temperatures on colony growth; (**H**) Effects of temperatures on sporulation. The letters indicate a significant difference between different treatments at the *p* < 0.05 level.

The fungal strain demonstrated growth and sporulation capabilities across all the tested conditions: 10 culture media, eight carbon sources, seven nitrogen sources, and temperatures ranging from 5 to 35 °C (Figure 4). Significant variations (*p* < 0.05) were observed in both the mycelial growth and sporulation yields among the different media types. All the tested media, except WHDA, supported mycelial development (Figure 4A), with CMA proving optimal for sporulation (Figure 4B). The carbon sources exerted significant differential effects (*p* < 0.05) on fungal development. D-sucrose maximized mycelial growth (Figure 4C), whereas D-lactose enhanced sporulation most effectively (Figure 4D). Among the nitrogen sources, D-peptone significantly outperformed the others (*p* < 0.05) for both hyphal expansion and spore production (Figure 4E,F). The optimal temperatures were 25 °C for mycelial growth and 30 °C for sporulation (Figure 4G,H).

### 3.6. Influence of LYZ0664 on Growth Indicators of V. unijuga

All six growth indices, plant height, root length, aboveground fresh weights, aboveground dry weights, underground fresh weights, and underground dry weights, exhibited a decline post inoculation. Specifically, inoculation with the pathogen significantly reduced the underground dry weight by 61.8%, the underground fresh weight by 39.0%, and the aboveground fresh weight by 33.7% (Table 2).

### 3.7. Influence of LYZ0664 on Photosynthesis Indicators of V. unijuga

**Figure 5:**
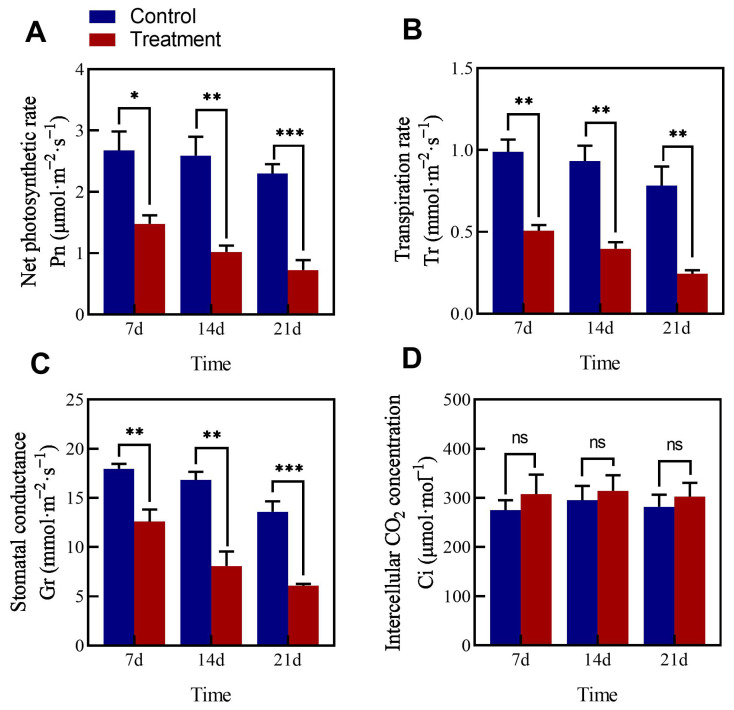
Effects of *Colletotrichum tofieldiae* on the net photosynthetic rate, transpiration rate, stomatal conductance, and intercellular CO_2_ concentration in *Vicia unijuga*. (**A**) Effects on the net photosynthetic rate; (**B**) Effects on the transpiration rate; (**C**) Effects on the stomatal conductance; (**D**) Effects on the intercellular CO_2_ concentration. * indicates a significant difference between the inoculated and control groups at the *p* < 0.05 level; ** indicates a significant difference between the inoculated and control groups at the *p* < 0.01 level; *** indicates a significant difference between the inoculated and control groups at the *p* < 0.001 level; ns indicates not significant difference between the inoculated and control groups.

Inoculation with the pathogen led to a gradual decline in the Pn, Gs, and Tr, with the respective values significantly reduced (*p* < 0.05) at all tested time points compared to the corresponding controls (Figure 5). Notably, the Pn and Gs showed the highest reductions on day 21 (68.77% and 55.15%, respectively; *p* < 0.001) compared to the controls. In contrast, inoculation with the pathogen increased the Ci at all time points but was not significantly different (*p* > 0.05) from that of the control plants.

### 3.8. Influence of LYZ0664 on Nutritional Quality of V. unijuga

**Figure 6:**
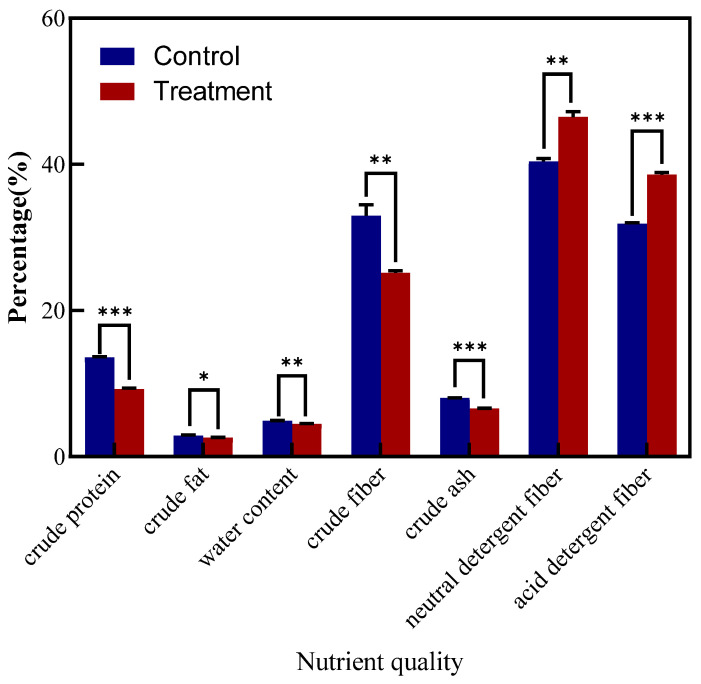
Influence on nutritional quality of *Vicia unijuga* inoculated with *Colletotrichum tofieldiae*. * indicates a significant difference between the inoculated and control groups at the *p* < 0.05 level; ** indicates a significant difference between the inoculated and control groups at the *p* < 0.01 level; *** indicates a significant difference between the inoculated and control groups at the *p* < 0.001 level.

Inoculation with the pathogen caused varying changes in the crude protein, crude ash, crude fat, neutral detergent fiber, acid detergent fiber, crude fiber, and water content (Figure 6), with significant differences compared to the controls. Highly significant differences (*p* < 0.001) were observed in the crude protein (13.6% to 9.3%), crude ash (8.0% to 6.6%), and acid detergent fiber (31.9% to 38.6%) contents among the tested samples.

## 4. Discussion

The pathogenic fungus *C. tofieldiae* was first identified as *Vermicularia tofieldiae* [42] based on its morphology and then later classified as a variant of *C. dematium* var. *minus* [43]. In 2009, Damm et al.’s work [36] strongly indicated that it belongs to the *C. destructivum* rather than the *C. dematium* clade, and renamed it *C. tofieldiae* based on the ITS sequences. It was subsequently classified within the spaethianum species complex [21]. The fungus exists on hosts as an endophyte, pathogen, and saprotroph [44]. It has been reported as a pathogen in *Dianthus* sp. in the UK [36], and in *Grevillea crithmifolia* and *Iris* × *germanica* in Australia [17]. In contrast, it has been found on dead stems and leaves in the form of saprophytic fungi on *Lupinus polyphyllus* in Germany [36], *Tofieldia calyculata* in Switzerland [45] and China [36,46], and *Ornithogalum umbellatum* in Japan [47]. Among the published reports, only Damm et al. [36] have detailed the morphological characteristics of the fungus, including the conidiomata, setae, conidia, and appressoria, among other characteristics. Noteworthy, these descriptions align with the findings of this study, supporting their validity

Despite the numerous species of *Colletotrichum*, the lack of distinctive morphological traits complicates their identification and classification [48]. For instance, the *C. spaethianum* clade contains only five species and lacks definitive morphological traits that serve as diagnostic markers [21]. Taxonomic studies of the *Colletotrichum* genus have demonstrated that certain species exhibit 100% homology in a single gene, resulting in erroneous identification outcomes [49,50]. Consequently, combined multi-gene tree construction has gradually become mainstream in research [51]. The spaethianum clade receives strong support in multi-locus analysis, which incorporates ITS, *GAPDH*, *CHS-1*, *HIS3*, *ACT*, and *TUB2* [21,36]. Herein, the fungal strains were identified using the ITS, *GAPDH*, *CHS-1*, *HIS3*, *ACT*, and *TUB2* genes for joint identification, which determined a 100% similarity to CBS 495.86 (*C. tofieldiae*). Consequently, the pathogen causing anthracnose in *V. unijuga* in Gansu Province, China, was identified as *C. tofieldiae* and was described as a novel disease globally.

There is a growing demand for food production and consumption, particularly for animal proteins [52]. This growing demand underscores the importance of the selection and breeding of high-yielding, nutrient-rich, and locally adapted forage varieties that are disease-resistant [53,54]. *V. unijuga* exhibits a diverse array of functions because of its high protein and soluble sugar content, which makes it an excellent forage grass suitable for cultivation [2]. Studies have postulated that *V. unijuga* is a promising candidate for domestication and promotion as a high-altitude forage species [55]. The appropriate field management of *V. unijuga* can enhance its aboveground dry matter and seed yield [27,28,56]. This study provides the first report on the effects of *C. tofieldiae* on the nutritional quality of host forage grasses. *C. tofieldiae* significantly compromised the nutritional quality of the forage grasses, reducing the crude protein, crude fat, crude ash, crude fiber, and water content. Of note, *C. tofieldiae* significantly reduced the crude protein content by 31.61%.

The existing studies have postulated that the transition between pathogenic and beneficial lifestyles of the same microbial species on the same host depends on the host and environmental conditions, rather than alterations in the microbial genome [57,58]. The evidence supporting this finding includes the symbiotic relationship between *Arabidopsis thaliana* and *C. tofieldiae*, which is exclusively activated under phosphate-deficient conditions [57,59]. Noteworthy, this interaction is particularly beneficial for Brassicaceae plants with limited phosphate absorption capabilities [57]. Conversely, transitions in symbiotic relationships is attributable to changes in microbial gene expression. Hiruma et al. [60] revealed that the activation of the ABA-BOT (ABA and the associated sesquiterpene metabolite botrydial) gene cluster in *C. tofieldiae* induces the transition of the fungus from a mutualistic to a pathogenic lifestyle. Zimmermann et al. [59] reported that the lack of a phosphorylated pathway of serine biosynthesis, particularly the major components, such as indolic glucosinolates, causes *C. tofieldiae* to behave like a pathogen rather than a beneficial microorganism in plants. Consistent with this, Díaz-González et al. [61] suggested that the strain Ct0861 significantly enhances the shoot length, weight, and yield of *Zea mays* and *Solanum lycopers* under optimal nutrient conditions. In summary, the role of *C. tofieldiae* as either a pathogen or a beneficial microorganism is contingent upon various factors, including environmental conditions and gene expression. This complex mechanism is a subject for further research. Photosynthesis serves as the origin of numerous beneficial substances [62] and is fundamental to nearly all life forms in plants. Enhancing plant photosynthesis is essential because of the limited agricultural land and increasing population [63]. Herein, *C. tofieldiae* infection significantly reduced the photosynthetic capacity of *V. unijuga*. The plants were cultivated in nutrient-rich soil without a phosphate deficiency. The decrease in the net photosynthetic rate, transpiration rate, and stomatal conductance in the infected plants indicates that the pathogenic behavior of *C. tofieldiae* is attributable to the activation of the ABA-BOT gene cluster. This activation potentially results in the production of abscisic acid (ABA), leading to stomatal closure.

*C. tofieldiae* has been documented as a pathogen in only three plant species, with reports limited to its morphological characteristics. This study is the first to report *C. tofieldiae* as a pathogen causing anthracnose in *V. unijuga* by utilizing Koch’s postulates, its morphological characteristics, and a multi-locus sequence analysis. The study also comprehensively analyzes the optimal medium type, carbon and nitrogen sources, and temperature conditions that promote the growth and sporulation of *C. tofieldiae*, which marks the first such investigation. Additionally, the host range of the pathogen is assessed. Moreover, the impact of *C. tofieldiae* on the growth, photosynthetic, and nutritional parameters of *V. unijuga* is evaluated for the first time. The findings of this study underscore the significant infective potential of *C. tofieldiae* and reveal its wide host range and non-host specificity. Nonetheless, further investigations on the epidemiology, distribution, host range [64], breeding of anthracnose-resistant *V. unijuga* varieties [65,66], and fungicide selection [67] should be conducted in controlled and natural ecosystems to develop effective control strategies aimed at preventing economic losses.

## 5. Conclusions

This study establishes *Colletotrichum tofieldiae* as the causal agent of anthracnose on *Vicia unijuga* in Gansu Province, China, representing the first global report of this pathogen–host interaction. Its identification was confirmed through Koch’s postulates, a morphological characterization, and a multi-locus phylogenetic analysis. We further delineated the optimal conditions for mycelial growth and sporulation, revealing the pathogen’s adaptability across diverse media, carbon and nitrogen sources, and temperatures. Notably, *C. tofieldiae* significantly compromised the host’s physiology and nutritional quality. The infected plants exhibited a reduced photosynthetic capacity and a 31.61% reduction in the crude protein content, critically diminishing its forage value. The host-range trials demonstrated non-specific pathogenicity, suggesting a broad infection potential.

## Figures and Tables

**Table 1:** Infection index of *Colletotrichum tofieldiae* on seven legumes.

Forage Species	Incubation Period (d)	Reseparation Rate (%)	Incidence Rate (%) (Mean ± SE)	Disease Index (Mean ± SE)
*Vicia unijuga*	4	65.00	60.00 ± 10.18 ^ab^	50.00 ± 2.89 ^a^
*Medicago sativa*	6	60.00	28.89 ± 4.44 ^b^	15.00 ± 5.00 ^bc^
*Onobrychis viciifoila*	4	53.33	40.00 ± 7.69 ^ab^	20.00 ± 6.67 ^bc^
*Vicia sativa*	3	76.00	73.33 ± 13.87 ^a^	57.22 ± 15.48 ^a^
*Astragalus adsurgens*	5	60.00	40.00 ± 11.55 ^ab^	15.00 ± 8.66 ^bc^
*Trifolium pratense*	3	80.00	60.00 ± 20.00 ^ab^	28.33 ± 8.33 ^b^
*Trifolium repens*	3	53.33	26.67 ± 6.67 ^b^	6.67 ± 1.67 ^c^

Different letters in the same column mean significant difference at *p* < 0.05.

**Table 2:** Influence of *Colletotrichum tofieldiae* on the growth of *Vicia unijuga*.

Measurement Index	Control	Treatment	Biomass Loss Rate (%)
Plant height (cm)	14.60 ± 0.81	10.8 ± 0.73	26.0
Root length (cm)	9.60 ± 0.40	8.20 ± 0.58	14.6
Aboveground fresh weights (g)	0.19 ± 0.02	0.13 ± 0.01	33.7
Aboveground dry weights (g)	0.05 ± 0.00	0.04 ± 0.00	19.1
Underground fresh weights (g)	0.24 ± 0.03	0.14 ± 0.02	39.0
Underground dry weights (g)	0.06 ± 0.00	0.02 ± 0.00	61.8

## Data Availability

All the sequences generated in this study were submitted to GenBank.

## Supplementary Materials

The following supporting information can be downloaded at: https://www.mdpi.com/article/10.3390/jof11080567/s1, Table S1: Sequences information for *Colletotrichum* spp. used in this study; Table S2: The standard of severity levels.
