# Supplementary material for: The Novel Disease Vicia unijuga Caused by Colletotrichum tofieldiae in China: Implications for Host Growth, Photosynthesis, and Nutritional Quality
Source: J Fungi (Basel). 2025 Jul 29;11(8):567. doi: 10.3390/jof11080567 (PMC12387466; doi:10.3390/jof11080567)
Supplement: Supplementary file 1 [file jof-11-00567-s001.zip › jof-3762145-supplementary.pdf]

Supplementary Tables

Table S1. Sequences information of *Colletotrichum* spp. used in this study (Damm et al. 2009).

Species	Strain	Host	GenBank Accession					
			ITS	*ACT*	*Tub2*	*CHS-1*	*GAPDH*	*HIS3*
Colletotrichum anthrisci	CBS 125334*	*Anthriscus sylvestris*	GU227845	GU227943	GU228139	GU228335	GU228237	GU228041
C. chlorophyti	IMI 103806*	*Chlorophytum* sp.	GU227894	GU227992	GU228188	GU228384	GU228286	GU228090
C. circinans	CBS 221.81*	*Allium cepa*	GU227855	GU227953	GU228149	GU228345	GU228247	GU228051
C. curcumae	IMI 288937*	*Curcuma longa*	GU227893	GU227991	GU228187	GU228383	GU228285	GU228089
C. dematium	CBS 125.25*	*Eryngium campestre*	GU227819	GU227917	GU228113	GU228309	GU228211	GU228015
C. fructi	CBS 346.37	*Malus sylvestris*	GU227844	GU227942	GU228138	GU228334	GU228236	GU228040
C. lilii	CBS 109214	*Lilium sp.*	GU227810	GU227908	GU228104	GU228300	GU228202	GU228006
C. lineola	CBS 125337*	*Apiaceae*	GU227829	GU227927	GU228123	GU228319	GU228221	GU228025
C. liriopes	CBS 119444*	*Lirope muscari*	GU227804	GU227902	GU228098	GU228294	GU228196	GU228000
C. rusci	CBS 119206*	*Ruscus sp.*	GU227818	GU227916	GU228112	GU228308	GU228210	GU228014
C. spaethianum	CBS 167.49	*Hosta sieboldiana*	GU227807	GU227905	GU228101	GU228297	GU228199	GU228003
C. spinaciae	CBS 128.57	*Spinacia oleracea*	GU227847	GU227945	GU228141	GU228337	GU228239	GU228043
C. tofieldiae	CBS 495.85	*Tofieldia calyculata*	GU227801	GU227899	GU228095	GU228291	GU228193	GU227997
C. tofieldiae	LYZ0664	*Vicia unijuga*	PP421934	PP426200	PP426197	PP426203	PP426206	PP426209
C. tofieldiae	LYZ0665	*Vicia unijuga*	PP421935	PP426201	PP426198	PP426204	PP426207	PP426210

C. tofieldiae	LYZ0666	*Vicia unijuga*	PP421936	PP426202	PP426199	PP426205	PP426208	PP426211
C. trichellum	CBS 118198	*Hedera sp.*	GU227813	GU227911	GU228107	GU228303	GU228205	GU228009
C. truncatum	CBS 151.35*	*Phaseolus lunatus*	GU227862	GU227960	GU228156	GU228352	GU228254	GU228058
C. verruculosum	IMI 45525*	*Crotalaria juncea*	GU227806	GU227904	GU228100	GU228296	GU228198	GU228002
C. lindemuthianum (outgroup)	CBS 151.28	*Phaseolus vulgaris*	GU227800	GU227898	GU228094	GU228290	GU228192	GU227996

CBS: Culture collection of the Centraalbureau voor Schimmel cultures, Fungal Biodiversity Centre, Utrecht, The Netherlands; IMI: Culture collection of CABI Europe UK Centre, Egham, UK; LYZ: isolations in this study; * ex-type and ex-epitype cultures.

Table S2. The standard of severity levels.

**Severity levels**	**Symptom**
0	Lesion-free, green stems
1	The diseased branches accounted for less than 25% of the total branches
2	The diseased branches accounted for 26% to 50% of the total branches
3	The diseased branches accounted for 51% to 75% of the total branches
4	The diseased branches accounted for more than 75 % of the total branches
